# Review of HIV-Related Cytopathology

**DOI:** 10.4061/2011/256083

**Published:** 2011-04-07

**Authors:** Tee U. Lang, Walid E. Khalbuss, Sara E. Monaco, Pam Michelow, Liron Pantanowitz

**Affiliations:** ^1^Department of Pathology, University of Pittsburgh Medical Center Cancer Pavilion, 5150 Centre Avenue, Suite 201, Pittsburgh, PA 15232, USA; ^2^Cytology Unit, Department of Anatomical Pathology, Faculty of Health Sciences, University of the Witwatersrand and National Health Laboratory Service, Johannesburg 2192, South Africa

## Abstract

Exfoliative and aspiration cytologies play a major role in the management of patients with human immunodeficiency virus infection. Common cytology samples include cervicovaginal and anal Papanicolaou tests, fine needle aspirations, respiratory specimens, body fluids, Tzanck preparations, and touch preparations from brain specimens. While the cytopathologists need to be aware of specific infections and neoplasms likely to be encountered in this setting, they should be aware of the current shift in the pattern of human immunodeficiency virus-related diseases, as human immunodeficiency virus patients are living longer with highly active antiretroviral therapy and suffering fewer opportunistic infections with better antimicrobial prophylaxis. There is a rise in nonhuman immunodeficiency virus-defining cancers (e.g., anal cancer, Hodgkin's lymphoma) and entities (e.g., gynecomastia) from drug-related side effects. Given that fine needle aspiration is a valuable, noninvasive, and cost-effective tool, it is frequently employed in the evaluation and diagnosis of human immunodeficiency virus-related diseases. Anal Papanicolaou tests are also increasing as a result of enhanced screening of human immunodeficiency virus-positive patients for cancer. This paper covers the broad spectrum of disease entities likely to be encountered with human immunodeficiency virus-related cytopathology.

## 1. Introduction

The current human immunodeficiency virus (HIV) pandemic has changed considerably, as infected people are now living longer with chronic HIV infection due to highly active antiretroviral therapy (HAART). With HAART therapy, the major cause of death in the later stage of acquired immune deficiency syndrome (AIDS) is malignancy, rather than infection. Moreover, the spectrum of malignancies encountered in HIV-positive patients has expanded to include both AIDS-defining cancers, such as cervical cancer, Kaposi sarcoma (KS), and non-Hodgkin's lymphoma (NHL), as well as non-AIDS-defining cancers (NADC), such as Hodgkin's lymphoma, anal cancer, lung carcinoma, and nonmelanotic skin cancer [1]. However, individuals unlikely to be on HAART therapy are still presented with opportunistic infections, such as tuberculosis, fungi, and parasites. HIV-infected patients have disproportionately high rates of infection with oncogenic viruses, such as human papillomavirus (HPV), Kaposi sarcoma human virus/human herpesvirus-8 (KSHV/HHV-8), and Epstein-Barr virus (EBV). HPV is closely linked to anogenital cancer [2–4] and HHV-8 associated malignancies include KS [5], primary effusion lymphoma (PEL) [6], and multicentric Castleman's disease [7]. Finally, EBV infection has been linked to Hodgkin's lymphoma [8], plasmablastic lymphoma (PBL) [9], and leiomyosarcoma [10]. 

**Figure 1:**
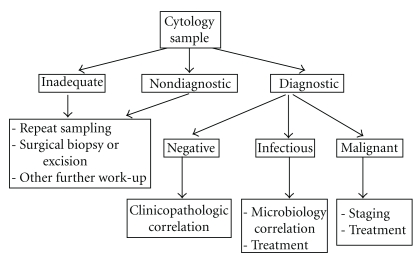
Proposed algorithm for the use of cytopathology in patients with HIV infection.

Both exfoliative and aspiration cytologies play a major role in the management of patients with HIV. Fine needle aspiration (FNA) using needles of variable sizes can be used for diagnostic purposes as well as a therapeutic modality, as in draining cystic lesions or effusions. FNA has gained much popularity in evaluating mass lesions in HIV-positive patients because it is noninvasive, well-tolerated, inexpensive, and avoids the necessity of subjecting patients to invasive and costly surgical excision [11]. In addition, an adequate FNA can usually provide sufficient material for ancillary studies, such as microbiology culture, flow cytometry, and/or a cell block for immunocytochemical stains. Anal Pap tests have now been employed at several centers to screen both men and women for possible anal cancer [12]. In some circumstances, cytology samples alone may not always be suitable. For example, the diagnosis of Castleman's disease may prove challenging by FNA alone. In such cases as in lymphadenopathy and bone and soft tissue lesions, a concomitant needle biopsy is recommended if resources permit. Another limitation is that FNA may not exclude all coexisting pathologies (e.g., a lymph node with lymphoma and Kaposi sarcoma).  Figure 1 is a schematic of the proposed role of cytology in the work-up of an HIV-positive patient. Cytopathologists will encounter HIV cytopathic effects, findings resulting from opportunistic infection, and/or morphology of neoplasia. This review covers all aspects of HIV-related cytopathology.

## 2. HIV Cytopathic Effect

HIV may induce a viral cytopathic effect on host cells [13]. This cytopathic effect was initially observed in vitro by investigators studying HIV-infected lymphocyte cultures [14]. Soon thereafter, researchers identified such viral particles in vivo, while studying HIV-infected human brain [15] and follicular dendritic cells in the lymphoid tissue [16, 17]. HIV causes two types of cytopathic effects: (i) single cell undergoing irreversible cell membrane ballooning, rupture, and lysis [13], and (ii) multinucleated giant cells formation via cell fusion involving the uninfected CD4 molecule [18]. The ability to lyse CD4 T cells contributes to the clinical condition AIDS. In cytology samples, multinucleated giant cells in lymphoid tissue resembling Warthin-Finkeldey cells are often evident in FNA of HIV-associated benign lymphoepithelial cyst-like lesions of the parotid gland [19]. The giant cells die soon after they are formed, further contributing to the depletion of CD4 T cells [18].

## 3. Cervical Neoplasia

Women infected with HIV have a high prevalence of HPV infection [20] and, therefore, are at increase risk of developing uterine cervical cancer [21, 22]. This can be explained by persistent HPV infection in HIV-seropositive women with multiple HPV subtypes, particularly high-risk HPV types [23, 24]. Depending upon the population studied, squamous intraepithelial lesion (SIL) is up to 40-fold higher in HIV-infected women [25], and up to 20% of these infected women are likely to develop cervical SIL within 3 years of their HIV diagnosis [26, 27]. Other studies have shown increased progression rates of low-grade SIL (LSIL) to high-grade SIL (HSIL) in HIV-positive women [28], and a relative risk of 5.4 for developing invasive squamous cell cancer (SCC) [3]. 

Cervical screening programs attempt to reduce the risk of cervical cancer by detecting early precursor lesion. A retrospective study by Maiman [25] demonstrated a higher rate of abnormal Pap test findings among HIV-positive women, reporting abnormalities in as high as 30–60% of Pap tests and evidence of dysplasia in 15–40% of Pap tests. The majority of these abnormal Pap tests are due to atypical squamous cells of unknown significance (ASC-US), found typically in women with a CD4 count <200  cells/mm^3^ [29]. The rate of abnormal Pap test is also higher in younger age groups for both LSIL and HSIL [30, 31]. Some studies have reported that Pap tests in the HIV population are not reliable in that they have high false-negative rates [27, 32]. However, not all studies support this finding [33]. Moreover, some studies have found lower sensitivity and specificity of HPV testing in HIV women compared to the general population [34]. This is not surprising given that many HIV-positive women are already HPV-positive, particularly with high risk HPV subtypes [35]. Hence, HPV testing may not be suitable in this population due to the high prevalence of HPV, including multiple types.

The combination of high false negative Pap tests and aggressive cervical disease progression have prompted new screening guidelines in HIV-positive women. Current guidelines from the Centers for disease control (CDC), American College of Obstetricians and Gynecologists (ACOG) and American Cancer Society (ACS) recommend annual Pap tests after initial HIV diagnosis, with an additional six month screen in the first year [36–38]. The CDC also recommends routine colposcopic screening in view of the high false negative Pap test in this setting. This recommendation is supported by the British Society for Colposcopy and Cervical Screening (BSCCS) guideline that advise all newly diagnosed HIV-positive women to have annual Pap and colposcopy tests [39]. However, alternate guidelines to perform routine colposcopy in all newly diagnosed HIV-positive women are required that are applicable to resource poor countries [34]. 

Current data suggests HAART has not significantly altered the rate of HIV-related cervical cancer [40]. However, there is some convincing evidence that the effect of HAART on cervical cancer may be most beneficial among women with early-stage HIV disease [4]. Recent FDA-approved HPV vaccination against low- and high-risk HPV has shown efficacy against persistent HPV infection. It remains to be seen whether early administration of the HPV vaccination will reduce the burden of HIV-related cervical neoplasia. The impact of vaccination may be limited in HIV-positive women due to the high diversity of oncogenic HPV seen in this population [41].

## 4. Anal Neoplasia

**Figure 2:**
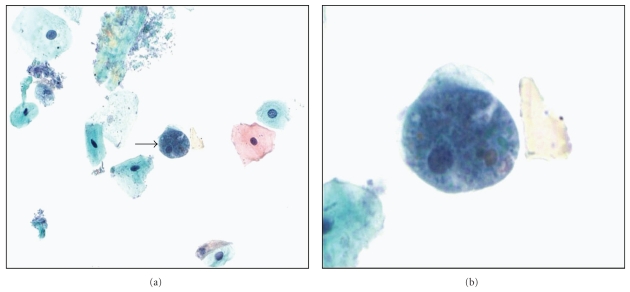
Anal Pap test showing (a) a trophozoite of *Entamoeba histolytica *(arrow, intermediate magnification) with (b) ingested erythrocytes (ThinPrep, Pap stain, high magnification).

The incidence of anal cancer has gradually increased over the last decade, notably in men who have sex with men [42]. In the USA, 2010 cancer statistics expect 5,260 new diagnoses and 720 deaths due to anal cancer [43]. Similar to cervical cancer, anal cancer is mainly associated with high-risk HPV infection acquired by HIV-positive individuals with low CD4 counts of <500/mm^3^ [44]. Although not widely established, screening with anal Pap tests has been shown to be a cost-effective screening method for the detection of SIL in HIV-infected patients [45]. The sensitivity of anal cytology to identify anal squamous lesions ranges between 69–93% [46], but it suffers from reported low specificity (32–59%) [47]. Moreover, there appears to be poor correlation between cytology-histology findings in these anal Pap tests [46]. Unlike cervical cancer, HPV testing of anal cytology samples has shown poor positive predictive value for high-grade anal intraepithelial neoplasia (AIN) [47], and it is recommended that all HIV patients with abnormal anal Pap tests (i.e., with a diagnosis of ASCUS and above) should undergo high-resolution anoscopy (HRA) with biopsy to detect high-grade AIN. Controversial issues concerning anal Pap screening that remain to be adequately addressed include screening frequency, timing to intervene with premalignant lesions, and algorithms for the management of anal Pap test results [48]. When interpreting anal Pap tests, cytopathologists should attempt to adhere to the 2001 Bethesda System utilized for reporting of cervical Pap tests [49]. A satisfactory anal-rectal specimen should have adequate cellularity with both nucleated squamous cells and rectal columnar cells. Other objects, such as obscuring fecal matter, herpes simplex virus infection and Candida spp., and unusual parasitic infections [50] (Figure 2) can be identified on anal cytology.

## 5. Skin and Soft Tissue Pathology

**Figure 3:**
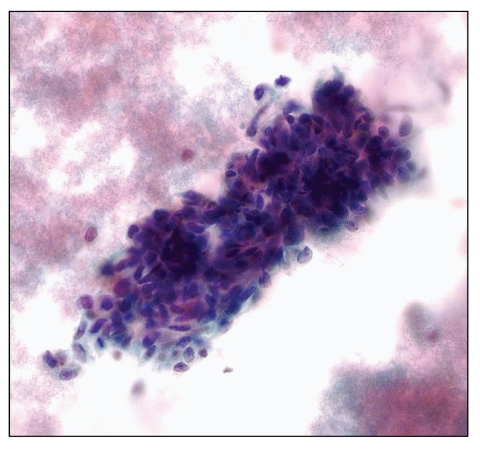
FNA of Kaposi sarcoma showing crowded spindled lesions with a bloody background (Pap stain, high magnification).

As many as 90% of HIV-positive patients may develop one or more skin diseases during the course of their illness [51]. The spectrum of skin diseases encompasses noninfective and infective dermatoses, adverse drug reactions, and dermatologic neoplasms. Kaposi sarcoma (KS) is the most common AIDS-defining cancer, which may involve both cutaneous and extracutaneous body sites. While most clinicians today perform biopsies for KS, FNA has previously been shown to be useful in the diagnosis of KS [52, 53]. Key cytologic features of KS (Figure 3) include cohesive clusters of spindle cells, a bloody background, and positive immunostaining of lesional cells with vascular (CD34) and lymphatic (D2-40) endothelium markers and/or the HHV-8 antibody latent nuclear antigen-1 (LNA-1). Although the demonstration of HHV-8 DNA by molecular techniques proves to be a useful adjunct in the diagnosis of aspirated KS [5], contamination of samples by mononuclear hematopoietic cells that harbor HHV-8 is possible and can be a misleading finding. 

**Figure 4:**
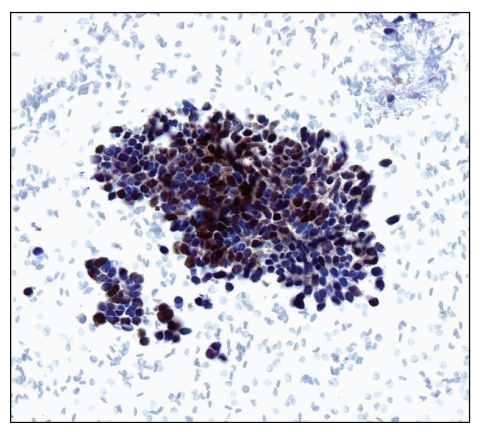
Merkel cell carcinoma cells in a cell block preparation are immunoreactive for Merkel cell polyomavirus (CM2B4 immunocytochemical stain, high magnification).

The incidence of skin cancers other than KS, such as SCC, basal cell carcinoma, and Merkel cell carcinoma (MCC), is increasing in HIV-positive patients [54, 55]. Dermatologic HPV infection in the HIV population can manifest as both anogenital lesions (e.g., condyloma acuminata and intraepithelial neoplasia) and nongenital skin diseases, such as epidermodysplasia verruciformis [56]. In addition, HIV-positive patients are predisposed to B-cell cutaneous lymphomas, often with an immunoblastic or centroblastic phenotype and CD30+ EBV+ immunophenotype [57]. HIV patients with these skin cancers tend to present at a younger age, manifest with multiple synchronous tumors, higher recurrence rate, treatment-associated complications, and a poorer prognosis compared to their HIV-negative counterparts [54]. MCC was recently shown to harbor a novel polyomavirus, Merkel cell polyomavirus (MCPyV), in the majority of cases [58, 59]. MCPyV can be detected in MCC using polymerase chain reaction (PCR) and the CM2B4 immunohistochemical stain (Figure 4).

Other rare soft tissue proliferations to be considered in the HIV-positive patient are mycobacterial pseudotumors and smooth muscle neoplasms. First described by Wood et al. [60], mycobacterial pseudotumors predominantly involve the lymph nodes. They are frequently associated with atypical mycobacteria, especially Mycobacterium avium intracellulare [61]. Therefore, for any spindle cell lesion in the setting of HIV infection, it is important to order an acid fast stain to see if these S100- and CD68-positive spindle cells contain mycobacterial organisms. A high incidence of EBV-related smooth muscle neoplasms including leiomyomas and leiomyosarcomas has been reported in various anatomic sites (mainly central nervous system, but also the gastrointestinal tract, liver, spleen, lung, adrenal, and skin) in HIV-infected adults and children [62, 63]. Detection of EBV (e.g., EBER in situ hybridization) along with smooth muscle markers (e.g., smooth muscle actin) may be of diagnostic aid.

## 6. Hematolymphoid Pathology

**Figure 5:**
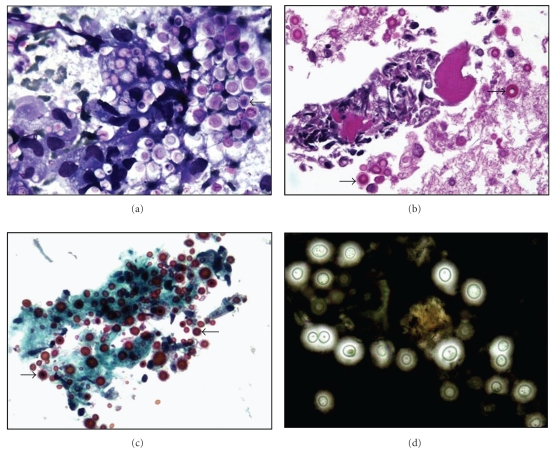
FNA of a neck lymph node cryptococcoma in a 38-year-old HIV-positive man shows multiple encapsulated yeasts (arrows) in the (a) direct smear (Diff Quick stain, high magnification) and (b) cell block (H&E stain, high magnification). (c) Encapsulated yeast stain positive with mucicarmine stain (high magnification) and (d) result in a clear halo with an India ink preparation (high magnification).

A large proportion of HIV-infected patients present with lymphadenopathy that is readily amenable to FNA. The etiologies differ by geographic region. In Western countries, such as the USA and England, reactive follicular hyperplasia is more common than infection or lymphoma [64–66] whereas infections, such as tuberculosis, prevail in developing countries [67, 68]. The cytomorphologic findings in reactive lymph node ranging from prominent follicular hyperplasia (specimens have abundant polymorphous lymphocytes and tingible-body macrophages) to aspirates derived from advanced-stage lymphadenopathy with involuted follicles (specimens are lymphoid depleted and rich in plasma cells). With the latter findings, Castleman's disease should be included in the differential diagnosis, and the invariably increasing branching hyaline capillary fragments should at least raise this diagnostic possibility. Although atypical follicular dendritic cells are thought to be diagnostic of Castleman's disease [7], they are not consistently found on FNA of lymph node [69]. Therefore, FNA is not recommended for the diagnosis of Castleman's disease [69], and a definite diagnosis will require biopsy, tissue examination, and immunophenotyping. Mycobacterial infection is a common infectious cause of lymphadenopathy in the HIV patient [64]. The cytologic findings of tuberculosis lymphadenitis may be granulomatous, necrotizing, or suppurative. The diagnosis can be confirmed by the demonstration of acid fast mycobacteria. In previously treated patients, culture of aspirated material may yield negative results, and PCR amplification may assist in such cases. Other rare infectious causes of HIV lymphadenopathy that can be diagnosed by FNA include pneumocystis [70], histoplasmosis [71], cryptococcus [72, 73] (Figure 5), and leishmaniasis [74].

The incidence of both Hodgkin and non-Hodgkin's lymphoma (NHL) is increasing in the HIV population. Hodgkin's lymphoma ranks among the most common NADC encountered, particularly the mixed cellularity and lymphocyte-depleted subtypes [75]. In HIV patients, Hodgkin's lymphoma may be widely disseminated with frequent extranodal disease, but rarely mediastinal involvement [76]. Between 75–100% of AIDS-associated Hodgkin's lymphoma cases have EBV infection. The diagnosis can be made by identifying Reed-Sternberg (RS) cells on FNA smears, along with confirmatory immunostains (e.g., CD30- and CD15-positive and LCA-negative RS cells). It is also estimated that 5–10% of HIV patients will develop NHL, including Burkitt lymphoma, plasmablastic lymphoma (PBL), primary effusion lymphoma (PEL), and HHV8-associated lymphoma arising from multicentric Castleman's disease. These are all aggressive, high-grade lymphomas that usually manifest with extranodal involvement [77]. Burkitt lymphoma accounts for 30% of all HIV-associated lymphomas, and EBV is positive in a subset of these malignancies. Diffuse large B-cell lymphomas, particularly the immunoblastic variant, can also be associated with EBV in HIV-positive patients. With the use of ancillary studies, such as flow cytometry, immunocytochemistry on cell block material, and FISH studies (e.g., for *myc* activation) on unstained slides prepared at the time of aspiration, the diagnosis of lymphoma can readily be made by FNA. 

**Figure 6:**
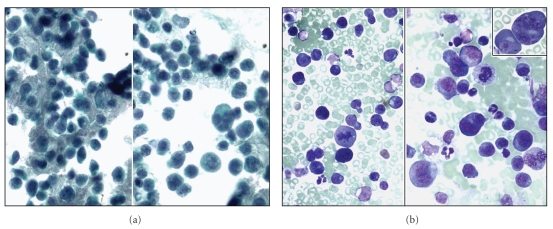
Atypical lymphoid cells in a case of primary effusion lymphoma of the pleura. (a) Pap stain (high magnification) and (b) Wright-Giemsa stain (high magnification).

**Figure 7:**
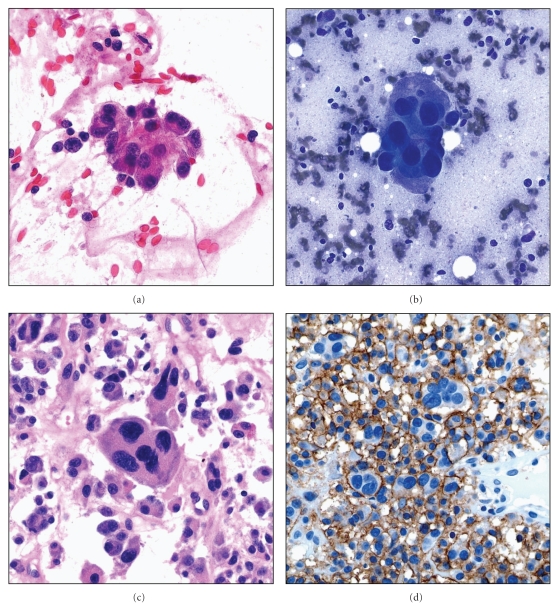
Anaplastic plasmacytoma of the pelvic bone showing atypical plasma cells with (a) Pap stain (high magnification), (b) Diff Quick stains (high magnification), and (c) in the cell block (H&E stain, high magnification). (d) Atypical plasma cells are immunoreactive with CD138 stain (high magnification).

PBL, initially described involving mainly the jaw and oral cavity of HIV-positive patients, has since been reported within lymph nodes and many different extranodal sites. Two subtypes of PBL have been described: (i) oral mucosa type and (ii) PBL with plasmacytic differentiation. Although this is a B-cell lymphoma, plasmablasts may be negative for CD45 (LCA) and the B-cell markers CD20 and PAX 5. However, these tumor cells are positive for CD79a, plasma cell antigens (CD138, CD38, and MUM-1), as well as CD56, CD30, and EMA. PBL is also closely associated with EBV [9], and, like Burkitt's lymphoma, may have structural alterations of the MYC locus. PEL, which represents only around 4% of AIDS-related lymphomas, is associated with HHV-8 and arises mainly with late-stage AIDS. Two subtypes of PEL are described: (1) classic PEL presents with effusions in pleural, peritoneal, and pericardial cavities, and (ii) solid PEL, which involves extracavitary tissue and may manifest with an effusion in addition to nodal or solid tissue lymphoma. The cytology of either PEL subtype is characterized by large atypical lymphoid cells ranging in appearance from immunoblastic to anaplastic phenotype (Figure 6) [78]. In the majority of cases, PEL cells lack B- and T-cell markers, but coexpress CD30, EMA, plasma cell antigens (CD38, CD138, and MUM-1), and HHV-8 [78]. PEL, like many of the other aggressive AIDS-related NHL, portends a poor prognosis [79]. Plasma cell neoplasia needs to be considered in lymphoma with plasma cell differentiation in the setting of HIV infection. HIV-positive patients are at increased risk of developing plasma cell neoplasia [80] at a much younger age, with more aggressive disease and often multiple plasma cell tumors in unusual locations. The cytomorphology of plasma cell tumors in HIV-infected persons often shows anaplastic features (Figure 7).

## 7. Pulmonary Disease

The lung is a rich niche for opportunistic infections, and, for that reason, respiratory samples from HIV patients are frequently examined. With HAART therapy,* pneumocystis* continues to be a major opportunistic pulmonary pathogen as well as extrapulmonary manifestations, such as the liver, spleen, skin, and pleural effusion in HIV-positive patients. A specific diagnosis of *pneumocystis* requires demonstration of organisms in respiratory specimens, and BAL is favored over induced sputum for *pneumocystis* investigation [81]. Sputum offers a specificity of nearly 100% and sensitivity of 55% [82] while BAL alone has a diagnostic yield of 97–100% [83]. In general, a more invasive procedure (e.g., BAL) used to collect specimens will provide better diagnostic yield. BAL and/or biopsy is preferable in difficult clinical settings, for example an uncooperative patient, a patient too dyspneic. In a patient with altered mental status, endotracheal aspirates can be submitted for *pneumocystis* investigation. The typical cytology finding shows foamy casts containing round or crescent cysts measuring 5–8 microns, with sporozoites located within these cysts. Numerous collapsed cysts may appear to be “budding,” and rarely a granulomatous response may be seen. Immunofluorescent staining with a monoclonal antibody directed against the *pneumocystis* cell wall of both cysts and trophozoites may increase the diagnostic yield in these cases [84], but this is not specific for *pneumocystis* [85]. Today, medical prophylaxis causes fewer organisms resulting in higher false-negative diagnoses for *pneumocystis* with cytology specimens [83]. 

**Figure 8:**
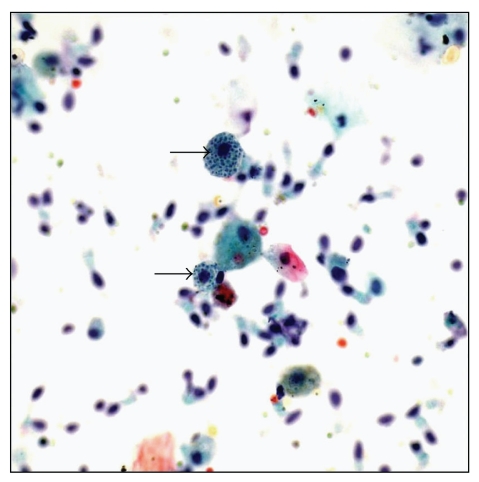
Bronchoalveolar lavage with histoplasma organisms (arrows) present within macrophages (ThinPrep, Pap stain, high magnification).

*Histoplasma capsulatum* causes pneumonia in HIV-positive patients resulting in a cavitary lesion, and lung mass that mimics malignancy or disseminated disease. *H. capsulatum* yeast forms (2–5 *μ*m in diameter) reside within macrophages (Figure 8), and will stain positively with Periodic acid-Schiff (PAS) and Gomori methenamine silver (GMS) stains. Mycobacterial infection of the lung in respiratory samples may be heralded by the finding of granulomatous inflammation with or without caseation [86]. A combination of an acid fast stain (e.g., Ziehl-Neelsen, Kinyoun, or auramine-rhodamine stains) with PCR in one study showed a sensitivity of 84% and a specificity of 100% [87]. Cytomegalovirus (CMV) is rarely found as a primary pulmonary pathogen in HIV-positive patients [88], in comparison to lung transplant recipients [89]. CMV is readily identifiable by viral inclusions in cytologic material obtained by BAL, and, if necessary, confirmed using immunocytochemistry [90].

Pleural effusions are commonly examined cytologic specimen in HIV-positive patients, and the prevalence among hospitalized AIDS patients is around 2–20% [91]. Common findings in this setting include inflammatory pleural exudates associated with a parapneumonic effusion, empyema, a lymphocytic exudate associated with mycobacterial infection, or malignancy due to lymphoma or metastatic carcinoma. Other rarer etiologies include volume overload, “crack cocaine abuse” which results in anthracotic pigmentation within effusion macrophages [92] and an idiopathic etiology. Bilateral pleural effusions appear to be more common in KS and lymphoma than in parapneumonic effusion [93]. KS may result in either a chylous effusion due to lymphatic obstruction or a bloody effusion with direct pleural disease. KS cells within pleural fluid tend to round up and take on an ameboid appearance, sometimes with erythrophagocytosis. AIDS-related lymphomas involving pleural effusions may be primary (i.e., PEL, referred to above) or secondary (i.e., disseminated systemic lymphoma).

## 8. Gastrointestinal Disease

Common gastrointestinal (GI) malignancies encounter in the HIV-positive patients include KS (60%), NHL (35%) [94], and anal neoplasia. KS has been reported to occur throughout the GI tract [94]. Involvement of the oral cavity by KS could be seen in 10–50% of cases [95, 96], and up to 75% of HIV patients with oral KS likely have concomitant GI lesions, frequently of their upper GI tract [97]. The GI system represents the second most common extranodal site for NHL, especially the anorectum [98]. The majority of these lymphomas are high-grade B-cell NHL that present with advanced tumor stage and life-threatening complications, such as ulcers, bleeding, bowel obstruction, and intussusception [99]. Low-grade MALT lymphoma of stomach is rare in HIV patients [100]. Nonneoplastic GI disease can accompany malignancy cases in HIV-positive patients. For example, CMV infection can cause GI perforation, obstruction, and pseudotumor formation [101]. Other infections also include cryptosporidiosis, microsporidiosis, Giardia, atypical mycobacteria, and amebiasis. When dealing with liver samples, viral hepatitis along with HIV can result in accelerated progression to hepatocellular carcinoma, with only a short interval between viral exposure and carcinoma development [102].

## 9. Head and Neck Pathology

HIV predisposes infected individuals to a broad spectrum of head and neck diseases. Due to a high density of lymphoid tissue, there is an increased risk of lymphoma in this region ranging from low-grade NHL (e.g., MALT lymphoma arising in the parotid gland) to aggressive NHL, such as plasmablastic lymphoma of the jaw. SCC is the third most common head and neck malignancy in HIV patients, after KS and NHL [103]. Synergy between HIV and HPV increases the prevalence of oropharyngeal cancer, especially tonsillar cancer [104]. The cytologic diagnosis of SCC is relatively straightforward, and p16 immunoreactivity of tumor cells may prove helpful in some cases. A unique feature is the presence of multinucleated tumor giant cells found in up to 98% of cases with HIV-associated SCC [105].

Benign oral ulcers and lesions may be attributed to infection such as herpes. Tzank smears can be used to render a rapid diagnosis, and it involves scrapping of an ulcer base to look for herpetic changes. The parotid gland in HIV-positive patients often contains bilateral cystic lesions referred to as benign lymphoepithelial cysts (BLEC). BLEC presents as painless swelling of the parotid, and cysts often recur after successful aspiration. FNA from these cysts yields clear to yellow-brown watery fluid, and cytologic findings comprise of admixed anucleated, superficial and intermediate squamous cells with lymphocytes, and tingible body macrophages [106]. Other disease entities arising within salivary glands in HIV-positive patients are low-risk lymphoma arising in BLEC, intraparotid lymphadenopathy, diffuse infiltrative lymphocytosis syndrome, and sialadenitis secondary to unusual infections, such as cytomegalovirus [107, 108] and cryptococcus [109]. Even the thyroid gland in HIV patients can rarely be involved by CMV infection [110] and pneumocystis [111].

## 10. Neuropathology

**Figure 9:**
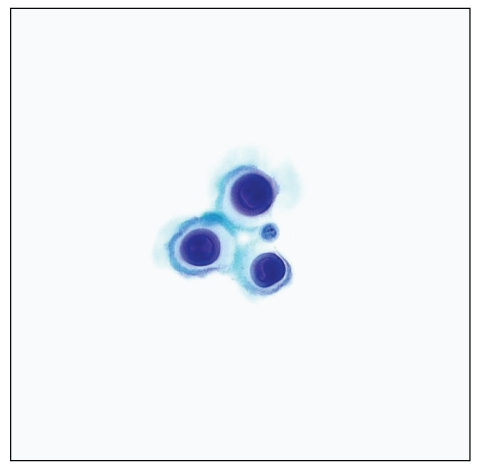
CSF showing cryptococcal yeast without inflammatory response (Pap stain, high magnification).

**Figure 10:**
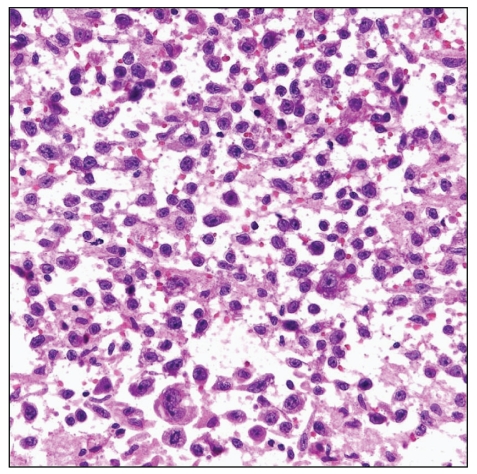
Intraoperative brain smear of a primary CNS diffuse large B-cell lymphoma (H&E stain, intermediate magnification).

HIV can affect the central (CNS) and peripheral nervous system by primary effect (e.g., HIV encephalopathy or polyneuropathy) or as a result of opportunistic infections or neoplasms. Benign lymphocytosis of the CSF is frequently encountered at the time of primary HIV infection [77], but other possible etiologies include viral encephalitis, infectious meningitis (e.g., tuberculosis and toxoplasmosis), neurosyphilis, and progressive multifocal leukoencephalopathy (PML). CSF is routinely examined in AIDS patients presenting with neurological symptoms for the identification of fungi, parasites, and lymphoma. Cryptococcal meningitis rarely causes a host inflammatory response, and yeast may range in number from very few to overwhelming numbers of organisms. Typical *Cryptococcus neoformans *in CSF are round (5–15 micrometer) with a thick refractile capsule and narrow based budding (Figure 9). Special stains used to identify encapsulated forms include an India ink preparation, GMS, PAS, and Fontana-Masson stains. Rarely, patients may have capsule-deficient cryptococci that may be easily mistaken for other microorganisms (e.g., histoplasmosis). Of note, CMV inclusions and *Toxoplasma gondii* are rarely seen in CSF [77]. However, *Toxoplasma gondii* may be observed in brain smears or touch preparation performed for an intracranial mass lesion. Definitive diagnosis in a brain smear requires identification of “bags of parasites” (i.e., cells infected with multiple bradyzoites) and smears usually also contain reactive astrocytes, mixed inflammatory cells, and background dirty necrosis. With HAART therapy, primary CNS lymphoma is infrequently encountered. These lymphomas are always high-grade B-cell lymphomas (Figure 10) that are strongly associated with EBV. They occur with advanced AIDS disease, and patients have a poor prognosis. A typical CSF specimen containing CNS lymphoma is usually highly cellular with large malignant cells showing prominent immunoblastic nucleoli. In some cases, detection of EBV in CSF may support the diagnosis of CNS lymphoma.

##  Conflict of Interests

The authors declare that they have no competing interests.

##  Disclosure

All authors of this paper declare that they qualify for authorship. All authors are responsible for the conception of this study, participated in its design and coordination, and helped to draft the paper. All authors read and approved the final paper.
